# Pharmacists' acceptability of a men's mental health promotion program using the Theoretical Framework of Acceptability

**DOI:** 10.3934/publichealth.2019.2.195

**Published:** 2019-06-27

**Authors:** Andrea Lynn Murphy, David Martin Gardner

**Affiliations:** 1 College of Pharmacy, Dalhousie University, 5968 College Street, PO Box 15000, Halifax, NS B3H 4R2, Canada; 2 Department of Psychiatry, Dalhousie University, 5909 Veterans' Memorial Lane, Halifax, NS B3H 2E2, Canada

**Keywords:** addictions, mental health, pharmacists, Theoretical Framework of Acceptability, qualitative

## Abstract

**Introduction:**

Community pharmacists are accessible, knowledgeable, and capable of providing mental health promotion and care in communities. This may not be a role that is recognized by the public, and men in particular. Differences between men and women exist in help seeking practices. Headstrong–Taking Things Head-On is a men's mental health promotion program for community pharmacies that was designed to increase the capacity of community pharmacists in caring for men with lived experience of mental illness and addictions. The program's core components included signage in pharmacies, education and training for pharmacists, and a website for use with patients.

**Methods:**

We applied the Theoretical Framework of Acceptability as the coding scheme to pharmacists' qualitative interviews to examine the acceptability of Headstrong for pharmacists.

**Results:**

Nine pharmacists consented to participate and all chose telephone interviews. With the exceptions of ethicality, affective attitude, and opportunity costs, all components from the TFA were coded in each of the nine transcripts. The most frequently coded constructs were perceived effectiveness of the intervention, burden, and self-efficacy. These were coded at least 20 times. The remaining categories ethicality, intervention coherence, affective attitude, and opportunity costs were coded between 11 to 17 times. Pharmacists' perceptions of the effectiveness of the program was mixed. The overall burden was perceived to be low, but opportunity costs appear to have limited the participation of some pharmacists in the program.

**Conclusion:**

Use of the Theoretical Framework of Acceptability as a coding scheme for qualitative data from community pharmacists in a men's mental health program was helpful for identifying issues with the program that may require redesign (e.g., signage). Program design should consider how services are advertised in the pharmacy setting, how personal values of pharmacists influence intervention coherence, and whether minimizing the burden of an intervention negates issues related to opportunity costs.

## 1. Introduction

Headstrong–Taking Things Head On (https://headstrong.life), hereafter referred to as Headstrong, is a program for men's mental health promotion. Headstrong is targeted to men in Nova Scotia, Canada, and was implemented in community pharmacies. Headstrong also has a standalone website (https://headstrong.life) that can be used by pharmacists and men with lived experience of mental illness and addictions. Headstrong focuses on six health topics: depression, anxiety, insomnia, alcohol use, tobacco use, and suicide. Overall, Headstrong is intended to build capacity in pharmacists and pharmacy teams so that they can help men access services, care, and community supports through the community pharmacy context. The core components of Headstrong included signage in the pharmacies to draw people to the pharmacy to ask about the program, an education program for pharmacists, and resources (e.g., Headstrong website, printed catalogue of mental health and addictions services and supports) for pharmacists to use with men. Signage included a 170 cm vertical mobile banner and a variety of shelf and counter signs. A training day for Headstrong was held to introduce and train pharmacists on the philosophy of the program and its resources. Additionally, pharmacists used an online learning management system that contained learning modules for each of the six areas of Headstrong and a module on motivational interviewing. The program's public website was designed and intended to support pharmacists and men in finding credible, curated resources for the content areas of Headstrong.

Pharmacists are accessible [1], trusted by the public [2]–[4], and have expanding roles in mental health care [5]. Headstrong in community pharmacies was designed as a means for reaching and engaging men about their mental health. Previous research has shown that men tend to avoid or delay help-seeking, particularly for emotional issues [6] and are less aware of health care services available to them [7],[8]. It has also been reported that women are more likely to seek health information face-to-face and online [9]–[11]. The Headstrong website was viewed as an integral component of the program for potential use by men, or for other people in men's lives, regardless of whether they directly interact with a pharmacist about the program. Accessing health information via the Internet can be convenient [12] and may serve to alleviate discomfort (e.g., embarrassment) experienced by some men, which has been associated with avoidance of help-seeking from general practitioners [13].

The expectations for enhanced program and service delivery by pharmacists in mental illness and addictions care is increasing [5]. Expectations of increased service delivery is not matched by the cumulative body of program implementation evaluation literature and there is much to be learned regarding pharmacists' experiences with mental illness and addictions program delivery and implementation [14]–[20]. Comparatively, there is more information available regarding pharmacists' experiences in other chronic disease management or health promotion programs (e.g., cardiovascular disease, asthma, immunizations) [14],[21]–[24]. This, in part, may be due to greater acceptance and comfort with physical health conditions (e.g., cardiovascular diseases) as compared to mental illnesses and addictions [25]. Although challenges and opportunities can exist with any new program or service, mental illness and addictions services present unique concerns in the pharmacy practice environment. Numerous issues (e.g., stigma, fear, privacy) can impact mental illness and addictions service delivery and patient outcomes. Whilst many factors will impact the implementation of community pharmacy-based services and programs, the overall successes and failures are fundamentally linked to pharmacists' behaviours in the practice environment. These behaviours are influenced by pharmacists' motivations, capabilities, opportunities, and the acceptability of the interventions to their users [26],[27]. As such, it is important to explore pharmacists' experiences with mental illness and addictions' health program implementation and delivery to support program revisions and future program designs. We applied the Theoretical Framework of Acceptability (TFA) [27] to better understand the acceptability of a men's mental illness and addictions program in community pharmacies.

## 2. Materials and method

### 2.1. Headstrong program theory and development

The development of Headstrong was underpinned by the Behaviour Change Wheel (BCW) [26]. We have used this theoretical foundation in other mental health programs [28],[29] to determine influences on pharmacists' and pharmacy staff behaviours as well as to design programs and services. The BCW was developed from frameworks of behaviour change and includes the behaviour system known as COM-B at the centre [26]. The COM-B includes capability (C), opportunity (O), and motivation (M), which ultimately interact to produce behaviours (B) [26]. The BCW includes nine intervention functions and seven policy categories that support intervention design [26]. The main Headstrong components align with the following intervention functions: education and training; persuasion; modelling; environmental restructuring; and enablement. These intervention functions were intended to encourage the desired behaviours of pharmacists interacting with men in the Headstrong program (e.g., recommending the website and community services).

### 2.2. Pharmacist interviews and data collection

All pharmacists (n = 31) of the 23 participating pharmacies were invited to participate in the Headstrong interviews. Consent was required for participation as per ethical requirements. The research coordinator notified pharmacists of the opportunity to participate in an interview to discuss the program. A research coordinator offered to conduct interviews via telephone or face-to-face. Interviews were audio-recorded, transcribed verbatim, and cleaned and anonymized by a research coordinator. The interview guide included questions such as, “Tell me about what the Headstrong program is to you?” and “Tell me about your ability to continue with/sustain elements of the Headstrong program as time went on?” The interview guide is available as an Appendix.

### 2.3. Research team and reflexivity

All interviews were conducted by the research coordinator who was completing a Masters of Public Administration at the time of the interviews and who had experience with qualitative research. The team leads (Murphy and Gardner) both have Doctor of Pharmacy degrees and are educators, researchers, and clinicians through our appointments with Dalhousie University. We identify as a woman (Murphy) and a man (Gardner). We have over 40 years in combined experience of clinical care and research in mental illness and addictions. We also believe in the importance of donating resources such as volunteering time and fundraising for non-governmental, community-based mental health organizations. A significant pillar in our program of research, in which we consistently use mixed methods, focuses on the design, implementation, and evaluation of programs, such as Headstrong and the Bloom Program [29], to enhance community pharmacists' care of people with experience of mental illness and addictions. As part of this process, we attempt to understand the experiences of pharmacists and other pharmacy team members and people with lived experience of mental illness and addictions who use pharmacists' services.

As per Finlay [30], reflexivity is a valuable tool used for many purposes including examining the impact of the position, perspective, and presence of the researchers. Prior to conducting the project, we engaged in reflexive practices in order to thoroughly examine our motivations and perspectives on the project. We decided that it would be important for the data to be collected by our research coordinator. This was intended to provide the opportunity for the pharmacist participants to feel more comfortable in answering and giving feedback about the program. Several of the pharmacists participating in Headstrong were our former students at our university pharmacy program. This inherently forces us to acknowledge that power imbalances were likely to exist and may continue to be perceived by participants. In data analysis, we discussed our own positioning as researchers but importantly as pharmacists with a significant knowledge base in mental illness and addictions care. For our purposes and our research, we discussed the potential influences from our experiential knowledge that could impact our interpretations and expectations of the data and the descriptions of practice within it. However, we also acknowledged that our experiential knowledge is also context-based and largely situated outside of community pharmacy practice.

### 2.4. Analysis

We used a directed content analysis technique with deductive coding [31]. The seven component constructs in the Theoretical Framework of Acceptability (TFA) [27] were used as the deductive coding scheme and the coding frequencies were presented as counts along with supporting quotes for each TFA component. The data were managed in NVivo 10 [32].

Coding was conducted individually by authors using the TFA. Each author followed a process of reading the transcript aloud once followed by re-reading the transcript and using line-by-line coding. Following coding of each transcript, the authors met to discuss their coding. We attempted to use one code from the TFA to isolate the meaning of the text versus double-coding passages of text.

### 2.5. Ethics

Ethics was received for the study from the Dalhousie University Research Ethics Board, file number 2015–3728. Written informed consent was obtained prior to the interviews. The signed consent forms were received from the pharmacists in advance of the interview in one of three ways: 1) a postal mailing of the signed consent form; 2) a facsimile of the signed consent form; or 3) a signed copy obtained via a face-to-face meeting.

## 3. Results

### 3.1. Participants

Nine pharmacists consented to participate in the interviews. All participants chose telephone interviews. Each interview began with a review of the project and the consent process. Interviews took 20 minutes on average.

### 3.2. Coding results

Four of seven TFA constructs were coded in each of the nine transcripts (Table 1). Coded in seven of nine transcripts were ethicality, affective attitude, and opportunity costs. The most frequently coded constructs were perceived effectiveness of the intervention, burden, and self-efficacy. These were coded at least 20 times and in all nine interviews (Table 1). The remaining categories ethicality, intervention coherence, affective attitude, and opportunity costs were coded between 11 to 17 times. Sample quotes are provided in Table 2 as well as through the description of findings for each TFA construct.

#### 3.2.1. Perceived effectiveness

The anticipated versus the experienced effectiveness of Headstrong was mixed for pharmacists. All nine pharmacists discussed that they or a colleague on staff at their pharmacy had used at least one resource from Headstrong (e.g., pamphlet, website, app) during the program. For many, they discussed successes with incorporating the Headstrong resources into their regular care and patient consultations as another tool to help people. For example, pharmacist 8 indicated, “Well, for me the two biggest things were anxiety and the smoking cessation. …I certainly was comfortable and glad to point them in the direction of those tools for either of those.” Pharmacist 2 also commented, “I refer a lot to the apps for anxiety and depression, especially TruReach and things like that.”

Several pharmacists indicated that the signage as a part of Headstrong did not produce the desired results and expressed disappointment that more people did not approach the pharmacists. Pharmacist 8 commented on the success with the resources but noted the issues with the signage, “I've referred patients to the website. …the postcards…and the brochure. We had the signage up. I'll be honest with you…we took the signage down probably a month ago. …Unfortunately it was in the way of some things, and management wanted it removed.” Similarly, pharmacist 3 stated, “My boss felt there was way too much actual stuff.” and that concern by management and staff was that “To the point that people just ignored it because there was just so much of it.” However, to contrast, pharmacist 1 indicated that other store staff were accepting of the signage and thought more could be of use and reflected on the placement, “we have signage located throughout the store.”

**Table 1.** Coding frequency in the component constructs of the Theoretical Framework of Acceptability [27] from pharmacists' interviews in the Headstrong program.

Theoretical Framework of Acceptability (TFA) constructs [27]	Definition [27]	Code frequency (number of interviews with code)
Perceived Effectiveness	Anticipated effectiveness: the extent to which the intervention is perceived to be likely to achieve its purpose.	38 (9)
Burden	Anticipated burden: the perceived amount of effort that is required to participate in the intervention.	26 (9)
	Experienced burden: the amount of effort that was required to participate in the intervention.	
Self-efficacy	The participant's confidence that they can perform the behaviour(s) required to participate in the intervention.	21 (9)
Ethicality	The extent to which the intervention has good fit with an individual's value system.	17 (7)
Intervention Coherence	The extent to which the participant understands the intervention and how it works.	16 (9)
Affective Attitude	Anticipated Affective Attitude: how an individual feels about the intervention, prior to taking part.	15 (7)
	Experienced Affective Attitude: how an individual feels about the intervention, after taking part.	
Opportunity Costs	Anticipated opportunity cost: the extent to which benefits, profits, or values must be given up to engage in the intervention.	11 (7)
	Experienced opportunity cost: the benefits, profits or values that were given up to engage in the intervention.	

**Table 2.** Theoretical Framework of Acceptability [27] illustrative quotes from pharmacists' interviews in the Headstrong program.

Theoretical Framework of Acceptability (TFA) constructs [27]	Illustrative quote
Perceived Effectiveness	“Well, helping patients or making them feel that they don't have to, you know, necessarily be alone. Like the nice thing about the Headstrong program was when you were counselling on a new medication or identifying a problem, and you could tell that the client was sort of shy to talk about it, you could be like, ‘Here's this great handout. You should check out this website. It's really narrowed down things for you. And, you know, you can do it in the comfort of your own home,’ and that sort of thing. So that was really great.” (Participant 7)
Burden	“But I think it just gives resources to potentially do a better job in some areas. And it doesn't take a lot of time. So I don't really see how it's not sustainable.” (Participant 5)
Self-efficacy	“I really liked the behavioural interviewing class or the presentation. I found that really helpful. Especially in the new year with like stop smoking and everything because we had a few patients coming up wanting to stop smoking. I found that really, really helpful. And I use it in my everyday practice for other things like blood pressure, cholesterol and all that kind of stuff too. So I did find that really helpful. And it kind of got me interested…. I did a couple more like CEs and courses just on the behavioural interviewing because I found that really helpful for just everyday practice.” (Participant 9)
Ethicality	“The girls, the other pharmacists, have mentioned it to a couple of people where they may not have before. Mentioned, you know, here's a resource that's available to you. But we're pretty darn good about mental health here. We advocate for people a lot, and we deal with a lot of people that are really marginalized [location]. So we're pretty darn good about it, if I do say so myself.” (Participant 3)
Intervention Coherence	“If we identify patients that are under any of those conditions that Headstrong provides resources for, then we can provide a resource to those patients, along with our counselling session. …We have signage located throughout the store. We have the Headstrong cards next to the counselling session. And we have the Navigator [resources catalog] on our desktop, as well as a hard copy for a resource for pharmacists to use if they need to or wish to.” (Participant 1)
Affective Attitude	“I think it's a natural fit for us because of our availability to the client. I think the more services that we can offer, the better.” (Participant 4)
Opportunity Costs	“The biggest deterrent I think would be if you're really busy, like overwhelmingly busy, and you know that doing that is going to take up x amount of time that you may not have. …But yeah, where I work by myself, it can get kind of overwhelming sometimes. You know, if you want to take 5 or 10 minutes to sit down and discuss something with a patient, but you don't really have that time.” (Participant 2)

#### 3.2.2. Burden

The coding for burden predominantly captured discussions of minimal efforts being required to participate in Headstrong. At least four of the pharmacists discussed pre-existing involvement with another mental illness and addictions program. Several additional pharmacists described a passion for advocacy and the desire to help those with mental illness and addictions and being “committed to these folks” (pharmacist 3). Pharmacist 2 expressed the sentiment discussed by many, “Both pharmacists [at pharmacy] are pretty open and helpful when it comes to, you know, working with patients with mental illness. I don't think there was a big change. It's just more tools at our fingertips, I guess, when working with that patient population. That's the biggest change.” The concept of burden in a negative connotation was evident when pharmacists brought forward issues with competing demands in the workplace. Pharmacist 8 discussed, “Workload is always an issue…. It's a very busy pharmacy. So it's sometimes hard to find the time. But again, just the nature of the program, it doesn't take a lot of time. …It's difficult to engage other…coworkers to participate in it. You know, I think that was on their end though, not so much my end or the program's end.”

#### 3.2.3. Self-efficacy

Self-efficacy by the pharmacists was expressed when they described learning, being comfortable in recommending the resources, and discussing examples of successfully helping men. Pharmacist 5 discussed being able to change their “style of engagement” and “the information I share” with patients as a result of participation. The program also encouraged pharmacist 9 to do additional training in motivation and behavioural interviewing and described, “I definitely have found that people, especially men, are more forthcoming and that quiet pauses in between aren't something that I need to fill. …I let them think. …And it definitely has made a difference in my practice. And even my boss said to me, …‘Why do people tell you things?’.”

#### 3.2.4. Ethicality

Ethicality was coded in seven transcripts and was coded when pharmacists discussed values from their personal and professional roles in practice. Pharmacist 5 indicated, “I think it's great. I think it's important. I think it's probably lacking. I think it's a good way to help the healthcare system where there seems to be a lot of people that maybe don't get the help they need or don't have the time with other healthcare providers that they're seeking.” However, there was also tension for at least three pharmacists regarding the gendered nature of the program that may have impacted their use. For example, pharmacist 3 indicated, “Quite frankly because it's for men only, that always has pissed me off from day one. …There's no reason for it to be a gendered thing. And if it became a non-gendered thing, I think I'd be a lot more comfortable saying, ‘Hey, we have this thing Headstrong. It's really great. There's a lot of really great information here.’ I just hate that it's for men only, to be quite frank.” Others, as demonstrated by pharmacist 2, discussed professional duties (e.g., responsibility) to patients, “I think it's our job. If there's something that you can do that helps your patients, I think it's your responsibility to do it. And these non-pharm things are as important, if not more, than the medications. So being aware that they exist and knowing about them, and being able to provide the information on them to your patients I think is part of our job and our duty.” Pharmacist 9 also discussed experiencing ethical dilemmas when considering resources that may help but may also create harms due to financial considerations with the more expensive apps on the website. They indicated, “And we just had a major shut down of [employer] where a lot of people were employed. …people are struggling. I don't want to put any more stress on people by saying, ‘Oh, yeah, try this app for $100.’ And they're like, ‘I can't even feed my kids this week, and that's why I need this app,’ to like de-stress or something.”

#### 3.2.5. Intervention coherence

All interviews had evidence of coding for intervention coherence. Pharmacists demonstrated clarity on the purpose of the program. However, in at least one example, the program may have been misinterpreted in term of the target demographic “I think a lot of the population that might benefit is very unstable.” (pharmacist 5). Pharmacist 9 described the purpose of the program and integrated their own personal example to demonstrate how it had worked, “So, the Headstrong program to me is just basically getting awareness out to men especially that it's okay that you talk about your feelings, and that it's not a bad thing, and that you don't have to keep it all in, and that there are resources for them, and that they're not alone. I get that a lot from patients. They don't want to tell anybody. Or, like they'll come in and they'll say that, you know, they've been suffering for 20 years and never told anyone. So, I think it's great that way.”

#### 3.2.6. Affective attitude

The majority of participants discussed positive feelings about the program. However, there were negative feelings that manifested in several ways that were brought out by the program. As mentioned, when the gendered nature conflicted with core values around inclusivity in practice, this created negative emotions for several pharmacists.

The activities that pharmacists engaged in during Headstrong were not part of a compensation model for community pharmacy. This brought to light issues with roles and compensation. Pharmacist 7 described, “I think there's a good spot for it [Headstrong program]. …Like the pharmacist is a very accessible person in the healthcare team. But as a pharmacist and as a manager who's trying to decide on staffing and that sort of thing, it's frustrating that the government doesn't support us or see us in that role even though we're doing that role. So, we're the most accessible healthcare professional, that you don't have to be triaged, and you don't have to have an appointment a lot of the times. And yet there's no compensation for that or no recognition in the payment model.”

#### 3.2.7. Opportunity costs

The opportunity costs expressed by pharmacists were often implicit versus directly stated and involved concerns around diverting time and money away from other tasks or removing human resources from services that generate revenue. Pharmacist 7 discussed concerns prior to attending the Headstrong training day as expressed to them from the pharmacy management, “At first they weren't really keen on it because it involved me being off-site for a day, and there was no fee for running the program here.” Pharmacist 9 indicated issues related to workload around workplace context issues and with other reimbursed interventions taking priority, “It was more difficult for us I think than maybe other stores because we were going through a renovation…. And with the flu shot season at the same time. So, I found it very hard. It's always hard during flu shot season to get any good interaction with patients because you're just so busy. …So it's hard, especially with flu shots, to actually use it. I wish it had been during a different time of year probably.”

## 4. Discussion

The Theoretical Framework of Acceptability was useful in determining several acceptability factors that may have influenced how pharmacists viewed and used the Headstrong program. These findings can be applied more generally for revising community pharmacy-based interventions and for improving their implementation. For example, when discussing Headstrong, the flexibility in using the resources for both men and women may not have been clear in our pharmacist participants as demonstrated by some comments from interviewees. This demonstrates that more work would be beneficial prior to intervention training in assessing participants' values, how these values align with respect to intervention coherence, and mechanisms to improve intervention coherence when values differ.

Importantly, it was noted when examining perceived effectiveness through the coding process that the signage related to the program may have been problematic in several locations. Pharmacists did not perceive that the signage created more inquiries in men's mental health care, which was disappointing for some. Others found issues with the gendered approach to the program and the signage. These factors may have also influenced how pharmacists felt towards the program (i.e., affective attitude), its effectiveness, and potentially impacted intervention coherence. The presence of the signage as a physical barrier as pointed out by some pharmacists is also important to note. Community pharmacies in the Canadian context are primarily retail spaces with high use by the public. Signage may impact advertising and potential sales for other items, traffic flow, and create other hazards (e.g., tripping, confusion for patients) in the pharmacy. Signage has been identified as an important factor as part of overall health literacy [33],[34] and safety [35] in the pharmacy context. Future use of signage in health promotion programs in the pharmacy context requires careful consideration of the practice environment including the size, the amount of signage required, and whether signage is effective to bring patients to discuss health issues with the pharmacists. Many pharmacy practice studies report on the utilization of signage as part of programs and services, such as with a sleep intervention by Tran et al. [35] in community pharmacies. However, researchers do not necessarily report on the effectiveness of the signage as part of the intervention. This makes comparisons with other interventions challenging given that this is an under-researched area.

The concern for the gendered signage brought forward important points regarding program designs and implementation and also the preparedness of pharmacists to engage with diverse groups in pharmacy practice. There has been more work in recent years to provide education to pharmacists for transgender and gender diverse individuals [36],[37]. This is especially important for our research area given the experience of mental illness and addictions, including suicide, by transgender and gender diverse people [37],[38]. Because of these experiences and feedback from participants, it provides us with an opportunity to explore various mechanisms to optimize program designs in the future. For example, at a minimum, we can start with using various tools and techniques such as those from Gender-Based Analysis Plus [39] when conceptualizing a research idea in a specific area to determine the potential impact on various groups.

In our study, pharmacists perceived minimal effort (i.e., burden) when participating in Headstrong. This may have been influenced by the fact that more than half of the sample discussed participation in mental illness and addictions programs at their pharmacies or identified as having a passion for the population, potentially signifying a more motivated group. However, issues with opportunity costs were also brought forward, which inherently could negate perceptions of low effort. Participants discussed issues with time, staffing, and competing demands in practice that would not allow them to consistently add Headstrong into their practice. For example, influenza vaccinations, which are a reimbursable service and seasonally increase workload for pharmacists, co-occurred during Headstrong delivery and took priority. The issue of lack of compensation for the Headstrong program was also directly brought forward by a pharmacist in our study. These findings regarding issues with time and incentives concur with those of others. For example, pharmacists from an Internet-based questionnaire in the US with specialty training in psychiatry also found barriers including time and issues with compensation [40]. A qualitative study of pharmacists in New Zealand with a special interest in mental health also supports concerns regarding time and compensation as barriers [41]. A survey of pharmacists in North Carolina similarly found time as a barrier for care by pharmacists to those with significant and persistent mental illness [42]. Based on our findings and those of others, it would be prudent to examine the relevance of commonly stated barriers (i.e., time restriction, lack of compensation) on pharmacists' performance when an intervention is perceived to be of limited burden.

### 4.1. Strengths and limitations

To our knowledge, this is the first study to use the Theoretical Framework of Acceptability to examine pharmacists' acceptability of a health promotion intervention in community practice. The analysis produced useful findings to consider for future iterations of Headstrong's design and implementation.

Headstrong pharmacists were a convenience sample of those who were interested in the program and wanted to participate. Only nine pharmacists from the 23 participating pharmacies consented to interviews therefore limiting the sample size and requiring caution for the interpretation of the results. This could indicate that volunteer bias is present in our overall sample of those wanting to offer Headstrong at their pharmacies and for those who wanted to participate in the interviews. We did not collect demographics from those who were interviewed given the small sample size of the program and the potential to identify those who participated in the interviews.

## 5. Conclusion

Use of the Theoretical Framework of Acceptability in evaluating the experiences of community pharmacists in a men's mental health program helped to identify issues with the program useful for informing changes in its design (e.g., use of signage). Pharmacists' perceptions of the effectiveness of the program was mixed and this may have potentially been impacted by public interest, values of participants, and opportunity costs in the face of competing services. Many pharmacists in the study had high self-efficacy for the service and were encouraged to change their practice and expand their professional development opportunities based on their involvement with the program. Design of programs should consider how services are promoted and advertised in the pharmacy setting, how personal values of pharmacists influence intervention coherence, and whether minimizing the burden of an intervention negates issues related to opportunity costs.
