# Supplementary material for: Pharmacists' acceptability of a men's mental health promotion program using the Theoretical Framework of Acceptability
Source: AIMS Public Health. 2019 Jun 27;6(2):195–208. doi: 10.3934/publichealth.2019.2.195 (PMC6606526; doi:10.3934/publichealth.2019.2.195)
Supplement: Supplementary file 1 [file publichealth-06-02-195-s001.pdf]

Public Health

AIMS Public Health, 6(2): 195–208.
DOI: 10.3934/publichealth.2019.2.195
Received: 04 March 2019
Accepted: 15 May 2019
Published: 27 June 2019

http://www.aimspress.com/journal/aimsph

Research article

Pharmacists' acceptability of a men's mental health promotion program using the Theoretical Framework of Acceptability

Andrea Lynn Murphy[1,*] and David Martin Gardner[2]

[1] College of Pharmacy, Dalhousie University, 5968 College Street, PO Box 15000, Halifax, NS B3H 4R2, Canada
[2] Department of Psychiatry, Dalhousie University, 5909 Veterans' Memorial Lane, Halifax, NS B3H 2E2, Canada

* **Correspondence:** Email: andrea.murphy@dal.ca; Tel: +19024941643; Fax: +19024941396.

Appendix: Semi-structured interview guide for Headstrong Taking Things Head-on
(REB #2015-3728; Version 2, April 12, 2016).

Tell me about what the Headstrong program is to you?

What elements of the Headstrong program have you used in your practice?

How easy or difficult has it been using things from the Headstrong program in your practice?

Do you know how to do things that were discussed in the modules for the Headstrong Program?

Tell us about any changes in your workplace towards people with mental illness that occurred with your participation in the Headstrong program.

Tell me about any changes in your role that have occurred as a result of participating in the Headstrong program.

What were your colleagues' and staffs' response to your participation in the Headstrong program?

How easy or difficult was it for you to engage in activities of the Headstrong program?

What problems did you encounter in doing things related to Headstrong?

Tell me about your ability to continue with/sustain elements of the Headstrong program as time went on.

What do you think the future will look like for yourself and the things you did with this program?

What's your comfort level in general with doing activities as described in the Headstrong program?

What has happened for you and your practice or business as a result of participating in the Headstrong program?

What are, or have been, the benefits of being in this program and engaging in activities related to Headstrong?

What are, or have been, the risks?

What has been your motivation in doing the Headstrong program?

What else do you want to achieve?

What are your beliefs about pharmacists engaging in activities as outlined in the Headstrong program?

When interacting with a person with mental illness that comes to the pharmacy, tell me about how the Headstrong program principles come into play. Are the principles of the Headstrong program something that is easily remembered to do as a part of patient care?

Tell me why you might decide not to do things related to the Headstrong program?

To what extent do physical or resource factors facilitate or hinder using principles or principles of the Headstrong program?

Are there competing tasks and time constraints?

Are the necessary resources available for you to undertake being a part of the Headstrong program or using Headstrong-related principles in your patient care?

To what extent do social influences facilitate or hinder you being a Headstrong pharmacist or using it in your care of patients?

Tell me about any mentorship that you saw occur during this program (i.e. have role models)?

In your opinion, are there any emotional factors, for example stress or mental workload, that facilitate or hinder your ability to engage in the Headstrong program?

What did you need to do to prepare to participate in the Headstrong program?

What changes occurred in the pharmacy to support the principles of the Headstrong program?

What kinds of changes in your work environment encouraged the use of the Headstrong program?

To what extent did your company/employer support you in using the Headstrong program?

Tell me about what you had to do differently in practice to participate in the Headstrong program?

Reflecting back to before training day and now, tell me about what's changed in your practice.

Tell me how, if at all, principles discussed in the Headstrong program are used in your practice.

What strategies have you used to make the Headstrong program principles part of your practice.

How often do you find yourself using principles of the Headstrong program?

How often do you reach out to your community members or other community-based organizations for people with lived experience for advice or guidance?

What's your opinion regarding the sustainability of Headstrong principles in your practice?

AIMS Press

© 2019 the Author(s), licensee AIMS Press. This is an open access article distributed under the terms of the Creative Commons Attribution License (http://creativecommons.org/licenses/by/4.0)
